# Proportional assist ventilation (PAV) versus neurally adjusted ventilator assist (NAVA): effect on oxygenation in infants with evolving or established bronchopulmonary dysplasia

**DOI:** 10.1007/s00431-020-03584-w

**Published:** 2020-01-25

**Authors:** Katie A. Hunt, Theodore Dassios, Anne Greenough

**Affiliations:** 1 Women and Children’s Health, School of Life Course Sciences, Faculty of Life Sciences and Medicine, King’s College London, London, UK; 2 The Asthma UK Centre in Allergic Mechanisms of Asthma, Kings College London, London, UK; 3 Neonatal Intensive Care Centre, King’s College Hospital NHS Foundation Trust, London, UK; 4 NIHR Biomedical Research Centre, Guy’s and St Thomas’ NHS Foundation Trust, King’s College London, London, UK

**Keywords:** Neurally adjusted ventilator assist, Proportional assist, Oxygenation index

## Abstract

Both proportional assist ventilation (PAV) and neurally adjusted ventilatory assist (NAVA) provide pressure support synchronised throughout the respiratory cycle proportional to the patient’s respiratory demand. Our aim was to compare the effect of these two modes on oxygenation in infants with evolving or established bronchopulmonary dysplasia. Two-hour periods of PAV and NAVA were delivered in random order to 18 infants born less than 32 weeks of gestation. Quasi oxygenation indices (“OI”) and alveolar-arterial (“A-a”) oxygen gradients at the end of each period on PAV, NAVA and baseline ventilation were calculated using capillary blood samples. The mean “OI” was not significantly different on PAV compared to NAVA (7.8 (standard deviation (SD) 3.2) versus 8.1 (SD 3.4), respectively, *p* = 0.70, but lower on both than on baseline ventilation (mean baseline “OI” 11.0 (SD 5.0)), *p* = 0.002, 0.004, respectively). The “A-a” oxygen gradient was higher on PAV and baseline ventilation than on NAVA (20.8 (SD 12.3) and 22.9 (SD 11.8) versus 18.5 (SD 10.8) kPa, *p* = 0.015, < 0.001, respectively).

*Conclusion*: Both NAVA and PAV improved oxygenation compared to conventional ventilation. There was no significant difference in the mean “OI” between the two modes, but the mean “A-a” gradient was better on NAVA.

**Table:** 

**What is Known:** • *Proportional assist ventilation (PAV) and neurally adjusted ventilatory assist (NAVA) can improve the oxygenation index (OI) in prematurely born infants.* *• Both PAV and NAVA can provide support proportional to respiratory drive or demand throughout the respiratory cycle.*	
**What is New:** *• In infants with evolving or established BPD, using capillary blood samples, both PAV and NAVA compared to baseline ventilation resulted in improvement in the “OI”, but there was no significant difference in the “OI” on PAV compared to NAVA.* *• The “alveolar-arterial” oxygen gradient was better on NAVA compared to PAV.*

**What is Known:**

• *Proportional assist ventilation (PAV) and neurally adjusted ventilatory assist (NAVA) can improve the oxygenation index (OI) in prematurely born infants.*

*• Both PAV and NAVA can provide support proportional to respiratory drive or demand throughout the respiratory cycle.*

**What is New:**

*• In infants with evolving or established BPD, using capillary blood samples, both PAV and NAVA compared to baseline ventilation resulted in improvement in the “OI”, but there was no significant difference in the “OI” on PAV compared to NAVA.*

*• The “alveolar-arterial” oxygen gradient was better on NAVA compared to PAV.*

## Introduction

Mechanical ventilation can be live-saving in the neonatal period but is also associated with complications such as bronchopulmonary dysplasia (BPD) and chronic respiratory morbidity [1]. Modes of ventilation that allow synchrony both of the timing and the level of support to the infant’s respiratory effort have been developed. Both proportional assist ventilation (PAV) and neurally adjusted ventilator assist (NAVA) provide support synchronised throughout the respiratory cycle. PAV delivers support proportional to the infant’s respiratory effort. Inspiratory pressure can be delivered in proportion to the change in flow (resistive unloading) and the change in tidal volume (elastic unloading), and the clinician can adjust the amount of unloading used [2]. The support delivered by NAVA is proportional to the electrical activity of the diaphragm, which is reflective of the neural respiratory drive. NAVA uses a specialised nasogastric tube with an electrode array at the distal end which detects the electromyogram of the diaphragm (Edi). The Edi is the signal used to trigger the ventilator and determines the level of support. The delivered pressure throughout each inflation is in proportion to the Edi signal. The clinician can adjust the NAVA level to increase or decrease the amount of pressure delivered per microvolt of Edi detected [3, 4].

The results of short-term studies [5–15] suggest that both PAV and NAVA improved oxygenation and were associated with lower airway pressures compared to conventional or other triggered modes of ventilation in prematurely born infants with evolving BPD, otherwise known as chronic pulmonary insufficiency of prematurity (CPIP) [16]. There are no studies in that population comparing PAV and NAVA, and hence the aim of this study was to compare the effect of PAV and NAVA on the oxygenation index in infants with evolving or established BPD. It has been demonstrated in an in vitro study that PAV was associated with a relatively long trigger delay [17]. In studies in adults, longer trigger delays have been found with PAV compared to NAVA [18, 19]. Hence, we hypothesised that NAVA would be associated with a superior (i.e. lower) OI in a crossover study as more of the infant’s respiratory cycle would be supported by pressure support.

## Methods

Infants were recruited from the tertiary neonatal unit at King’s College Hospital NHS Foundation Trust between June 2017 and July 2018. Infants were eligible for the study if they were born at a gestational age of less than 32 weeks and remained ventilated at or beyond 1 week after birth, that is, they had evolving or established BPD [20]. Those receiving neuromuscular blockade or with complex congenital cardiac abnormalities were excluded. The study was approved by the London-South East NHS Research Ethics Committee and the Health Research Authority and prospectively registered on clinicaltrials.gov with the identifier NCT02967549. Written informed parental consent was obtained.

Infants were routinely ventilated via shouldered Coles endotracheal tubes (Portex, Smith Medical, Hythe, UK) which have been shown to have minimal to no leak [21]. Prior to entry into the study, the infants were ventilated using SLE 5000 or 6000 ventilators (Specialised Laboratory Equipment, Croydon, UK) in assist control (A/C) or synchronised intermittent mandatory ventilation (SIMV) modes. Volume targeting was preferentially used, with target tidal volumes between 5 and 7 ml/kg as per the Unit’s guidelines. Some clinicians, however, preferred to use pressure-limited ventilation. During the study, the infants were transferred to either the Servo-n ventilator (Maquet Critical Care, Solna, Sweden) or the Stephanie ventilator (Stephan GMBH, Gackenbach, Germany) depending on whether they had been randomised to receive first NAVA or PAV. The ventilator circuits were changed according to ventilator type. The randomisation sequence was generated with a random number generator and allocations concealed inside opaque, sealed and consecutively numbered envelopes. Infants received ventilation at their baseline settings on either ventilator, followed by 2 h of either NAVA or PAV. At the end of the 2-hour period, a blood gas was taken, and the infant was transferred to the other study ventilator, following which the above sequence was repeated, that is, 1 h on their baseline settings and 2 h on the study mode. Infants were monitored throughout, and the heart rate, respiratory rate and oxygen saturations were recorded every 10 min, and all desaturations less than 88% were documented.

NAVA was delivered by the Maquet Servo-n ventilator. A six French Edi catheter of appropriate length for the infant’s weight (49 cm for 500–1500 g, 50 cm for 1000–2000 g) was inserted oro- or nasogastrically prior to the commencement of the study and left in place until the study was completed. The Edi catheter was positioned as per the manufacturer’s guidance using the specialised tape measure in the packaging and correct position confirmed using the Edi catheter positioning guide function on the ventilator (Maquet Servo-n User Manual Version 1.1). The Edi was monitored using the Servo-n ventilator throughout the entire study, including on baseline settings, and whilst PAV was delivered via the Stephanie ventilator.

During NAVA, the NAVA level was set by observing the displayed pressure waveform on the ventilator during three to five breaths, whilst the infant was ventilated using their baseline settings and then adjusted so that the pressure on NAVA closely matched that delivered on baseline settings, aiming for a peak Edi of between 5 and 15 μV. The apnoea time was set to 2 s to avoid hypoventilation and the Edi trigger to 0.5 μV as recommended by the manufacturer for neonatal use. The positive end-expiratory pressure (PEEP) was set at the same level as during baseline ventilation. Maximum PIP was initially limited to 5cmH_2_O above that set on baseline settings, as the Servo-n ventilator will not deliver pressures more than 5cmH_2_O below this limit. Ventilator parameters were manually collected with a single value that was expected to represent the existing stable baseline.

PAV was delivered using the Stephanie ventilator (Stephan GMBH, Gackenbach, Germany). Whilst ventilating on baseline settings, the infant’s compliance was recorded from the ventilator display every 10 min. The mean of the six results was calculated to determine the degree of elastic unloading required to return the infant’s compliance to normal, that is, 2 ml/cmH_2_O [22]. Initial elastic unloading was set at 75% of that value and increased to 100% after 10 min if no abnormal waveforms were observed [17]. No resistive unloading was used as this can result in oscillation in the airway pressure waveform, which may inhibit diaphragm activity [23, 24]. The PEEP was set as the same level as during baseline settings.

The FiO_2_ was adjusted to maintain saturations between 92 and 96% as per the Unit’s guidelines. Expiratory tidal volume, FiO_2_, respiratory rate, MAP, PIP and PEEP were recorded from the ventilator display (no PIP or PEEP is displayed during PAV). Blood gas analysis was performed using capillary samples. Capillary samples were not formally arterialised. Blood gas samples were analysed immediately using the blood gas analyser on the neonatal unit (ABL90 Flex, Radiometer, Brønshøj, Denmark).

As capillary blood samples were used, we calculated quasi OIs and alveolar-arterial oxygen gradients which we have designated “OIs” and “A-a” gradient, respectively. The primary outcome, “OI”, was calculated as (MAP*FiO_2_*100)/(partial pressure of oxygen (pO_2_). Secondary outcomes included the “A-a” gradient calculated as (FiO_2_*(Patm – pH_2_O) – (PCO_2_/0.8)-pO_2_), where Patm = 101.33 kPa and pH_2_O = 6.3 kPa; the oxygen saturation to fraction of inspired oxygen ratio (SF ratio) calculated as SpO_2_/FiO_2_ and the PO_2_/FiO_2_ ratio. In addition, the peak and tonic Edi during each epoch were obtained from the Maquet ventilator.

BPD was diagnosed as oxygen dependency for at least 28 days and as mild, moderate or severe depending on support required at 36 weeks corrected gestational age as per the NIH consensus guidelines [25].

### Sample size

A sample size of 18 infants was chosen as this would allow detection of one standardised difference in the results with 80% power at the 5% significance level. In a previous study [14], we reported a difference in the mean OI of infants supported by NAVA compared to ACV was greater than 3 with a standard deviation of the infants’ results being 2. We had also reported a difference of 3 in previous studies of PAV [6, 8]. Thus, the calculated sample size would be able to detect such a difference (i.e. > 3) between the results on the two modes.

### Statistical analysis

Results were compared from the end of each period of PAV, NAVA and conventional ventilation. Data were assessed for normality using a Shapiro-Wilk test. Normally distributed data were analysed using a one-way repeated measures ANOVA with Bonferroni correction. If the assumption of sphericity was violated, a Greenhouse-Geisser correction was applied. Non-normally distributed data were analysed using a Friedman test. Statistical analysis was carried out using IBM SPSS Statistics version 25 (IBM Corporation, Armonk, New York, USA.)

## Results

**Fig. 1:**
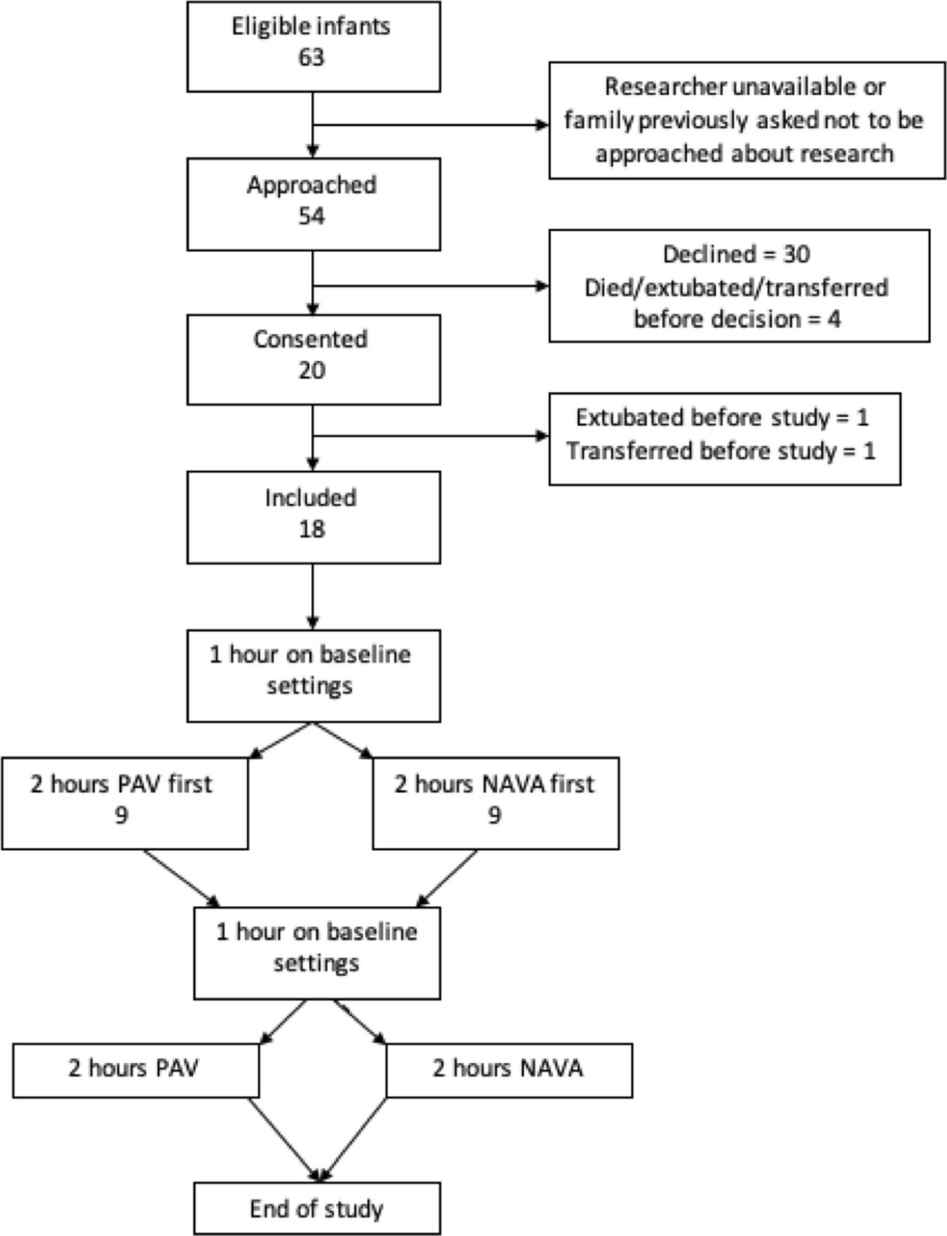
Consort diagram of recruitment and study protocol

Eighteen infants were studied (Table 1) (eight male, ten female). They were born at a median gestational age of 25.3 (range 23.6–30.3) weeks with median birthweight of 750 (range 454–950) grams. They were studied at a median of 20.5 (range 8–58) postnatal days. All of the infants were subsequently diagnosed with BPD; 13 had severe BPD, 3 moderate and 2 mild. Prior to the study, 15 infants were receiving volume-targeted ventilation, with target tidal volumes between 5 and 7 ml/kg; the others were receiving pressure-limited ventilation. Nine infants were randomised to receive NAVA first (Fig. 1). NAVA levels used ranged between 0.4 and 1.8.

**Table 1:** Baseline demographic details

N	18
Sex (male)	8
Gestational age (weeks)	25.3 [23.6–30.3]
Birthweight (grams)	750 [454–950]
Postnatal age at study (days)	20.5 [8–58]
Weight at study (grams)	865 [700–1800]

Results are presented as median [range]

**Fig. 2:**
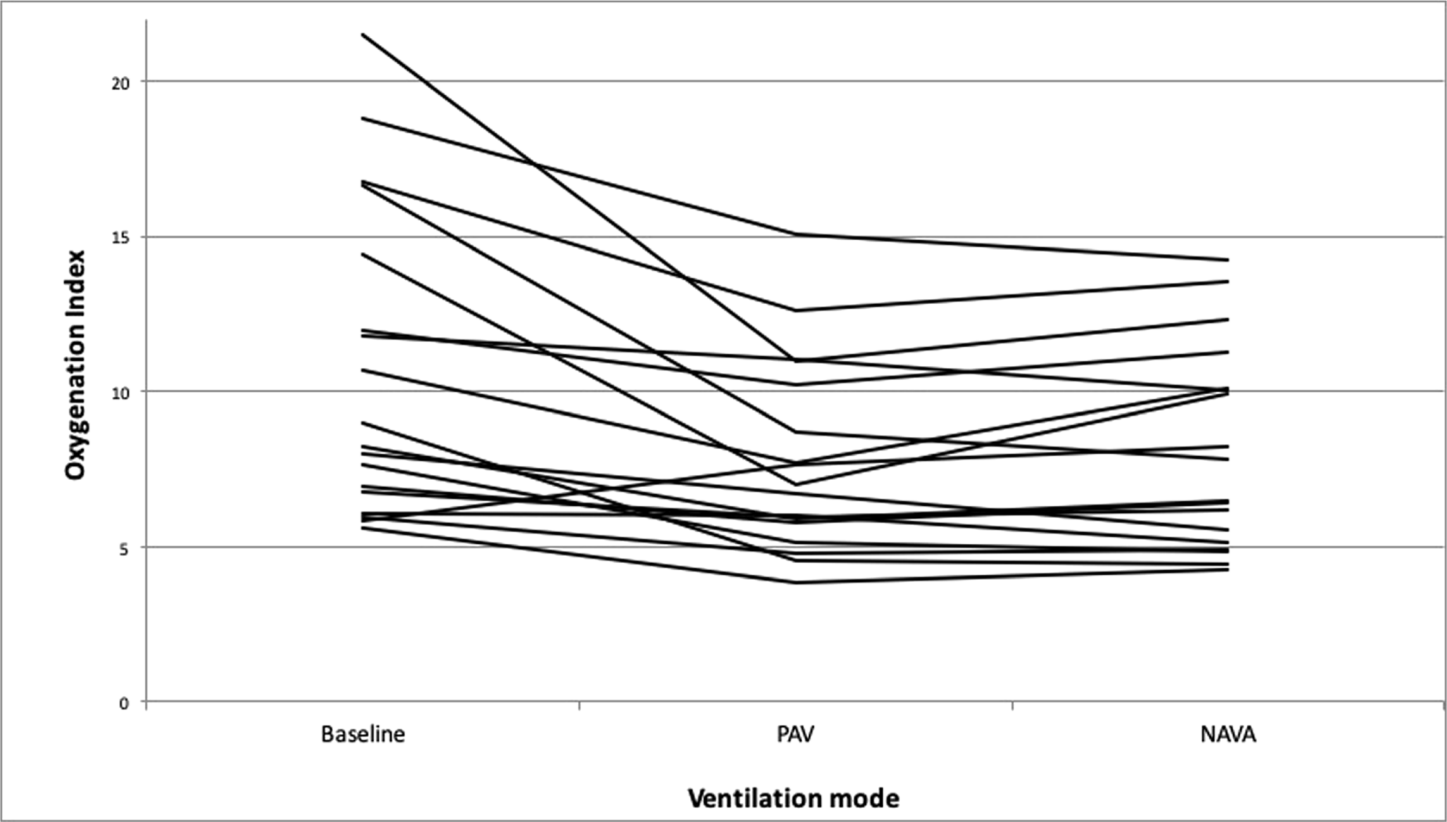
“Oxygenation index” of individual infants shown by linked data points on baseline ventilation, PAV and NAVA

There was no significant difference in the mean “OI” at the end of 2 h on PAV compared to on NAVA (mean 7.8 (standard deviation (SD) 3.2) versus 8.1 (SD 3.4), *p* = 0.7), but on both PAV and NAVA, the mean “OI” was significantly lower than on baseline ventilation (Table 2, Fig. 2). The FiO_2_ was higher on PAV than on NAVA (mean 0.39 (SD 0.15) versus 0.37 (SD 0.13) *p* = 0.024) and the MAP lower on PAV than on NAVA (8.3 cmH_2_O (SD 1.1) versus 9.0 cmH_2_O (SD (0.9), *p* = 0.025)) (Table 2). If only those 13 infants who went on to develop severe BPD were included, the “OI” was significantly lower on NAVA or PAV than at baseline (8.4, 8.8 vs 12.8, *p* = <0.001, *p* = 0.023, respectively), but the “OI” was not significantly different on PAV and NAVA (*p* = 0.29).

**Table 2:** Comparison of ventilatory, blood gas and diaphragmatic electrical activity parameters between baseline ventilation, PAV and NAVA

	Baseline	PAV	NAVA	p	Pairwise comparisons
“OI”	11.0 [5.0]	7.8 [3.2]	8.1 [3.4]	0.001	n-p 0.70 b-p 0.002 b-n 0.004
Mean airway pressure (cmH_2_O)	9.5 [1.1]	8.3 [1.1]	9.0 [0.9]	<0.001	n-p 0.025 b-p < 0.001 b-n 0.02
FiO_2_	0.40 [0.15]	0.39 [0.15]	0.37 [0.13]	<0.001	n-p 0.024 b-p 0.28 b-n 0.003
“A-a” gradient (kPa)	22.9 (11.8)	20.8 (12.3)	18.4 (10.8)	<0.001	n-p 0.015 b-p 0.127 b-*n* < 0.001
SpO_2_/FiO_2_	268 [96]	268 [92]	295 [102]	0.002	n-p 0.04 b-p 1.0 b-n 0.04
PO_2_/FiO_2_	104 (35)	120 (40)	126 (42)	0.001	n-p 0.79 b-p 0.033 b-n 0.002
PCO_2_ [kPa]	8.1 [1.7]	8.9 [1.8]	8.8 [1.7]	0.026	n-p 1.0 b-p 0.007 b-n 0.16
PO_2_ [kPa]	5.4 [0.76]	5.66 [0.68]	5.6 [1.0]	0.69	
Respiratory rate (breaths/min)	61 [9]	66 [8]	56 [9]	0.001	n-p 0.003 b-p 0.19 b-n 0.093
Expiratory tidal volume (ml/kg)	6.2 [0.7]	6.4 [1.0]	6.5 [1.2]	0.32	
Peak inspiratory pressure (cmH_2_O)*	17.8 [3.5]		14.3 [3.0]		b-*n* < 0.001
Positive end-expiratory pressure (cmH_2_O)*	5.6 [4.7–7]		6 [5–7]		b-n 0.94
pH	7.31 [0.67]	7.29 [0.68]	7.3 [0.66]	0.053	
Peak electrical activity of the diaphragm (microvolts)	13.6 [5.8–40.4]	13.1 [2.4–38.4]	11.3 [5.2–28.5]	0.33	
Minimum electrical activity of the diaphragm (microvolts)	2.2 [0.9–4.2]	2.3 [0.7–4.2]	2.4 [0.7–4.5]	0.92	

Results are presented as mean (standard deviation) or median [range]

n-p = comparison between NAVA and PAV; b-p = comparison between baseline and PAV

b-n = comparison between baseline and NAVA

*Data unavailable for two infants

The “A-a” gradient was significantly higher on PAV than on NAVA (20.8 versus 18.4, *p* = 0.015.) It was also significantly lower on NAVA than at baseline (18.4 versus 22.9, *p* < 0.001), but there was no statistically significant difference between PAV and baseline (20.8 versus 22.9, *p* = 0.127) (Table 2). If the 13 infants who went on to develop severe BPD were analysed separately, both PAV and NAVA were associated with lower “A-a” gradients compared to baseline ventilation (23.2 (SD 13.7) and 20.9 (SD 11.4) versus 26.5 (SD 12.1) kPa, *p* = 0.031, 0.001, respectively), but there was no statistically significant difference between the results on PAV and NAVA (*p* = 0.18). The SpO_2_/FiO_2_ ratio was lower on PAV and baseline than on NAVA (*p* = 0.004) (Table 2). There was no significant difference in the PO_2_/FiO_2_ between NAVA and PAV, but both were higher than at baseline (Table 2).

There were no significant differences between the expiratory tidal volumes or the pCO_2_ levels on PAV compared to on NAVA (Table 2). There were no significant differences between base excess or HCO_3_ results on PAV and NAVA. The PCO_2_ was statistically significantly higher on PAV than at baseline (8.9 vs 8.1 kPa, *p* = 0.016). The respiratory rate was significantly higher on PAV than on NAVA. The peak and minimum Edi were not significantly different between modes of ventilation (Table 2).

## Discussion

We have demonstrated that there was no significant difference in mean “OI” between PAV and NAVA, but both modes resulted in a reduction in “OI” compared to baseline conventional ventilation. We used the “OI” as the primary outcome as this gives a summary of the effectiveness of each ventilator mode compared to baseline. The mean airway pressure, however, was lower and the FiO_2_ higher on PAV than on NAVA. As the “OI” is a composite of the MAP and FiO_2_, then those differences being in the opposite direction resulted in similar “OIs”. The lower MAP on PAV resulted in a higher FiO_2_ requirement to maintain the same level of oxygenation. The lower MAP on PAV might reflect lower levels of synchrony, a different shape to the airway pressure waveform and/or a longer trigger delay [17–19]. The significantly higher “A-a” gradient (i.e. worse) on PAV compared to NAVA is consistent with the higher FiO_2_ requirement, as is the significantly lower SpO_2_/FiO_2_ (ie worse). The PO_2_/FiO_2_ was lower on PAV, but this did not reach statistical significance.

Both PAV and NAVA have been compared to conventional and other triggered modes of ventilation in small short-term studies in infants. PAV as compared to pressure-controlled conventional ventilation has been shown to reduce the OI in infants with respiratory distress syndrome [5] and to reduce the OI, peak and MAP and the work of breathing (as measured by PTPdi) in infants with evolving or established BPD [6–8]. Although there have been no randomised controlled trials of PAV in neonates, in adults, PAV versus pressure support ventilation resulted in a shorter duration of intensive care days [9]. NAVA has also been shown to have advantages over conventional ventilation in prematurely born infants. NAVA compared to pressure control ventilation and to pressure-limited synchronised intermittent mandatory ventilation (SIMV) in crossover studies, including those where infants had longer durations on each mode, resulted in lower peak inspiratory pressures (PIP) and a reduction in the work of breathing [10–13]. In prematurely born infants with evolving or established BPD, NAVA compared to pressure control ventilation in a crossover study resulted in reduction in the OI, MAP, FiO_2_ and PIP [14]. There has been one randomised controlled trial of NAVA, which was compared to pressure control ventilation and enrolled infants born between 28 and 36 + 6 weeks gestational age with acute respiratory distress. There was no significant difference in the primary outcome of the duration of ventilation (35 versus 26 h, *p* = 0.21). There was also no difference in the incidence of BPD, but the study was not powered to detect this [15].

To our knowledge, there have been no previous studies in the neonatal population that have compared NAVA and PAV, but a study in adults compared NAVA and PAV to baseline pressure support ventilation [19]. The OI was not reported, but when targeting the same tidal volumes, the PIP and MAP were higher on NAVA than on PAV with comparable blood gases, although the PaCO_2_ was statistically significantly higher on PAV than on NAVA, as was also found in our study. The small increase in PaCO_2_ may be due to the way that NAVA and PAV switch to back up ventilation after an apnoea period or to the amount of unloading during PAV and the NAVA level set. If either were to be used for longer periods, then careful monitoring and adjustment of settings as required would be advised.

There are strengths and some limitations to our study. We calculated the “OI” and “A-a” gradients from capillary samples, but this was a crossover study, so each infant acted as their own control. Thus, whilst arterial samples may have been more precise, we suggest that use of capillary blood samples would not have substantially influenced our results. We did not use arterialisation of the capillary samples as there is controversy in the literature as to whether this improves the correlations with arterial results. Furthermore, capillary blood gases have been shown to correlate well with arterial gases if infants are stable and not cardiovascularly compromised [26], particularly at lower levels of arterial oxygenation as in our cohort [27]. Additionally, it has been shown that in infants with congenital diaphragmatic hernia, indices of oxygenation obtained from capillary blood gas samples and changes in these indices over time predict survival, thus suggesting that capillary oxygenation is a valid method of monitoring clinical status [28]. Nevertheless, for future studies, transcutaneous monitoring of carbon dioxide and oxygen levels could be considered. We did not assess the infants’ comfort levels, and this plus assessment of the degree of asynchrony would be useful in future studies.

The NAVA level was set as per the manufacturer’s guidelines. Using the manufacturer’s guidelines, the MAP level during NAVA was decreased compared to baseline setting suggesting that the level was appropriate. All of the infants in our study either had or were subsequently diagnosed with BPD, indicating that our inclusion criteria successfully identified those with evolving or established BPD. The wide range of postnatal ages in our study population may have influenced our results, but advantages of PAV and NAVA over conventional ventilation have been demonstrated in infants with a variety of postnatal ages in previous studies. Targeted tidal volumes of between 5 and 7 mls/kg were used, as studies have shown chronically ventilated infants may require 7 mls/kg [29, 30] and in the latter study (30) to reduce the work of breathing. It could be suggested that in babies with BPD 2-hour epochs may not be sufficient to demonstrate changes in oxygenation. Previous studies, however, have demonstrated changes in oxygenation using both modes for shorter epochs of 45–60 min [5, 6, 14]. Indeed, we were able to demonstrate improved oxygenation over baseline settings for both PAV and NAVA using 2 h epochs and differences in the two modes with regard to the A-a gradient. In one study [12], 12-h periods were used, but the order was not randomised. Although there were no significant differences in blood gases between NAVA and pressure-regulated volume control (PRVC), neural apnoeas were only seen during PRVC, and less fentanyl was administered during NAVA (12).

In conclusion, we have found no significant differences in the “OI” after 2 h of support by PAV or NAVA but both significantly improved the OI compared to baseline ventilation. Other indices, as the ‘A-a’ gradient and SF ratio demonstrated that NAVA improved oxygenation over PAV, but the differences were relatively small.

## Abbreviations

A-a: Alveolar-arterial gradient
BPD: Bronchopulmonary dysplasia
CPIP: Chronic pulmonary insufficiency of prematurity
Edi: Electromyogram of the diaphragm
FiO_2_: Inspired oxygen concentration
MAP: Mean airway pressure
NAVA: Neurally adjusted ventilatory assist
OI: Oxygenation index
PAV: Proportional assist ventilation
PEEP: Positive end-expiratory pressure
PIP: Peak inspiratory pressure
PRVC: Pressure-regulated volume control
VTe: Expiratory tidal volume
